# Supplementary material for: The Potential Use of Fungal Co-Culture Strategy for Discovery of New Secondary Metabolites
Source: Microorganisms. 2023 Feb 12;11(2):464. doi: 10.3390/microorganisms11020464 (PMC9965835; doi:10.3390/microorganisms11020464)
Supplement: Supplementary file 1 [file microorganisms-11-00464-s001.zip › microorganisms-2195808-supplementary.pdf]

Supporting Information for

The Potential Use of Fungal Co-Culture Strategy for Discovery of New Secondary Metabolites

Shuang Xu, Mengshi Li, Zhe Hu, Yilan Shao, Jialiang Ying and Huawei Zhang *

School of Pharmaceutical Sciences, Zhejiang University of Technology, Hangzhou 310014, China;
* Correspondence: hwzhang@zjut.edu.cn

Contents

TableS1 Fungal－fungal co-culture derived new SMs (**1**－**109**) S2

TableS2 Fungal－bacterial co-culture derived new SMs (**110**－**151**) S6

TableS3 Fungal－host co-culture derived new SMs (**152**－**158**) S8

References S9

Table S1 Fungal–fungal co-culture derived new SMs (**1**–**109**).

Number	Co-culture Strains	Source	Co-culture Medium	Biological Activity	Reference
**1**<br>**2**<br>**3**<br>**4**<br>**5**	*N. oryzae* and *B. bassiana.*	Seeds of *Dendrobium officinale* collected from Wenshan, Yunnan Province, China	PDB	Compounds **1** and **2** showed significant nitric oxide (No) inhibitory activity.	[1]
**6**<br>**7**<br>**8**<br>**9**<br>**10**<br>**11**<br>**12**<br>**13**	*Armillaria* sp. MK079569 and *Epicoccum* sp. YUD17002	Rhizomes of the underground portion of *Gastrodia elata* collected from a plantation field at Xiaocaoba, Zhaotong, Yunnan Province, China	PDB	Compound **13** showed moderate i*n vitro* cytotoxic activities and weak acetylcholinesterase (AChE) inhibitory activity.	[2]
**14**<br>**15**<br>**16**<br>**17**<br>**18**	*N. oryzae* and *I. lacteus*	Seeds of Dendrobium officinale collected from Wenshan of Yunnan Province, China	PDB	Compound **18** showed anti-AChE activity.	[3]
**19**<br>**20**<br>**21**	*Pleosporales* sp. *F46* and *A. pilosum F47*	Pedicel of the medicinal plant *M. fortunei,* which was collected from Qingdao, China	PDB		[4]
**22**<br>**23**<br>**24**<br>**25**<br>**26**	*Phoma* sp. YUD17001 and *Armillaria* sp.	Rhizomes of *Gastrodia elata* collected from a plantation field at Xiaocaoba, Zhaotong, Yunnan Province, China	PDB	-	[5]
**27**<br>**28**<br>**29**<br>**30**<br>**31**<br>**32**	*Armillaria* sp. and *Phoma* sp. YUD17001			-	[6]
**33**<br>**34**<br>**35**<br>**36**<br>**37**<br>**38**<br>**39**<br>**40**	*P. fuscum* and *P. camembertii/clavigerum*	A single sample of surface water from Berkeley Pit Lake	PDB	Compound **33** exhibited antimicrobial activity	[7]
**41**	*T. harzianum* M10 and *T. pinophilus* F36CF	*T. harzianum* strain M10 was isolated from composted hard wood bark *T. pinophilus* F36CF was from a secondary branch of a strawberry tree collected in the isle of Favignana	PDB	Cytotoxic activity	[8]
**42**<br>**43**<br>**44**<br>**45**<br>**46**	*A. nidulans* and *E. dendrobii*	-	PDB	-	[9]

**47**<br>**48**<br>**49**					
**50**	*P. lilacinum* and *B. cinerea*	*B. cinerea* was isolated from a sick cucumber leaves. *P. lilacinum* was isolated from tomato roots	PDB	-	[10]
**51**<br>**52**<br>**53**	*P. orientoasiaticus* KMRB19040316 and *X. flaviporus* KMRB19080638	Korea Mushroom Resource Bank at Seoul National University	PDB	Compound **5** displayed antibacterial effect on *S. aureus*.	[11]
**54**	*Nigrospora* sp. and *Stagonosporopsis* sp.	*Nicotiana tabacum* L. (No. Y20210917) in Yuxi of Yunnan Province, China	PDB	Antifungal activities	[12]
**55**<br>**56**	*P. ostreatus* SY10 and *P. eryngii* SY302	Shandong Province Key Lab of Applied Mycology in China	Liquid medium (glucose 10 g/L KH_2PO_4 1 g/L, $MgSO_4$ 0.5 g/L, peptone 2 g/L, 1L sterilized wate)		[13]
**57**	*C. sambuci* FT1061 and *E. sorghinum* FT1062	A healthy fruit of the plant Rhodomyrtus tomentosa collected on the Big Island in Hawaii	Liquid medium (mannitol 20 g/L, sucrose 10 g/L, monosodium glutamate 5 g/L, KH_2PO_4 0.5 g/L, $MgSO_4 \cdot 7$ H_2O 0.3 g/L, yeast extract 3 g/L, corn steep liquor 2 mL/L, 1 L distilled water)	-	[14]
**58**	*P. pinophilum* FKI-5653 and *T. harzianum* FKI-5655	Soil collected in Hachijo Island, Tokyo, Japan	Glucose–peptone broth	-	[15]
**59**	two *Aspergillus alliaceus* strain	A marine alga	Malt liquid media (malt extract 10 g/L,yeast extract 4 g/L,glucose 4 g/L)	Cytotoxic activity	[16]
**60**	*Chaunopycnis* sp. CMB-MF028 and *T. hamatum* CMB-MF030	Inner tissue of a marine pulmonate false limpet *Siphonaria* sp. collected at the rocky intertidal zone of Moora Park, Shorncliffe, Queensland	ISP2 broth	-	[17]
**61**<br>**62**<br>**63**<br>**64**<br>**65**<br>**66**	*A. sclerotiorum* SCSGAF 0052 and *P. citrinum* SCSGAF 0053	Gorgonian *Muricella flexuosa* collected from the South China Sea, Sanya, Hainan Province, China	Liquid medium (glucose 10 g/L, solublestarch 10 g/L, $MgSO_4$ 1 g/L, KH_2PO_4 1 g/L, peptone1 g/L, sea salt 30 g/L)	Compound **65** showed cytotoxic activity.	[18]
**67**<br>**68**	*T. versicolor* and *G. applanatum*	Shandong Province Key Lab of Applied Mycology, China	Liquid medium (glucose 10 g/L, KH_2PO_4 1 g/L, $MgSO_4$ 0.5 g/L, and peptone 2 g/L 1L sterilized water)	-	[19]
**69**	*Aspergillus* sp. FSY-01 and FSW-02	A rotten fruit of mangrove Avicennia marina in Zhanjiang, Guangdong Province, China	Medium GYP (glucose 10 g/L, yeast extract 1 g/L, peptone 2 g/L, crude seasalt 3.5 g/L)	-	[20]
**70**<br>**71**	strains Nos. 1924 and 3893	An estuarine mangrove in Hong Kong, China	Liquid medium (glucose 10 g/L, peptone 2 g/L, yeast extracts 1 g/L, crude marine salt 3.5 g/L, running water 1 L)	Antibacterial activities	[21]
**72**	*Phomopsis* sp. K38 and *Alternaria* sp.	South China sea coast	Liquid medium (glucose 10 g/L,	Compound **72** had weak	[22]

	E33.		peptone 2 g/L, yeast extract 1 g/L, NaCl 30 g/L)	cytotoxic activity against Hep-2 and HepG2 cells.	
**73**	*Phomopsis* sp. K38 and *Alternaria* sp. E33.		Liquid medium (glucose 10 g/L, peptone 2 g/L, yeast extract 1 g/L, NaCl 30 g/L)	Antimicrobial activity	[23]
**74**<br>**75**	*Phomopsis* sp. K38 and *Alternaria* sp. E33.		liquid medium (glucose 10 g/L, Peptone 2 g/L, yeast extract 1 g/L, NaCl 30 g/L).	Antifungal activities	[24]
**76**	*N. oryzae* and *B. bassiana*	Seeds of Dendrobium officinale from Wenshan, Yunnan Province, China	Rice medium	Antifungal activity against *B. bassiana* and *N.oryzae*	[25]
**77**<br>**78**<br>**79**<br>**80**<br>**81**<br>**82**	*Pestalotiopsis sp.* and *P. bialowiezense*	*Pestalotiopsis* sp. was isolated from the leaves of tea tree, collected from the Enshi Autonomous Prefecture of Hubei Province, China. *P. bialowiezense* was isolated from the fresh soft coral Sarcophyton subviride, collected from the Xisha Island in South China Sea	Rice medium	Compound **77** showed significant β-glucuronidase inhibitory potency.	[26]
**83**<br>**84**	*A. sydowii* EN-534 and *P. citrinum* EN-535	Fresh tissue of the marine red alga Laurencia okamurai collected from Qingdao, China	Rice medium	-	[27]
**85**<br>**86**	*P. bilaiae* MA-267 and *P. chermesinum* EN-480.	*P. bilaiae* MA-267 was isolated from the rhizosphere of the marine mangrove plant *Lumnitzera racemosa* that was collected at Hainan Island. *P. chermesinum* EN-480 was isolated from the fresh tissue of marine red algal *Pterocladiella tenuis*, collected from Shandong province, China	Rice medium	Potent inhibitory activities against *Ceratobasidium cornigerum* and *Edwardsiella tarda*.	[28]
**87**	*A. terreus* EN-539 and *P. lilacinus* EN-531	Inner tissues of the marine red alga *Laurencia okamurai*	Rice medium	Inhibitory activity against *A. brassicae*, *E. coli*, *P. piricola*, and *S. aureus*	[29]
**88**<br>**89**<br>**90**<br>**91**<br>**92**	*A. sulphureus* KMM 4640 and *I. felina* KMM 4639	*I. felina* was isolated from marine sediments collected at a depth of 10 m,The South China Sea, coast of Vietnam. *A. sulphureus* KMM 4640 was isolated from marine sediments	20 g of rice, 20 mg of yeast extract, 10 mg of KH_2PO_4, and 40 mL of natural sea water	Compound **89** has cytotoxic activity.	[30]
**93**<br>**94**<br>**95**	*A. carneus* KMM 4638 and *I. felina* KMM 4639	*A. carneus* was isolated from superficial mycobiota of the brown alga *Laminaria sachalinensis* collected on Kunashir Island *I. felina* was isolated from marine sediments collected at a depth of 10 m, Van Phong Bay, the South China Sea,	20 g of rice, 20 mg of yeast extract, 10 mg of KH_2PO_4, and 40 mL of natural sea water	-	[31]

		Vietnam			
**96**<br>**97**	*P. influorescens* and *P. nobilis*	Sediment samples obtained from the Windebyer Noor, Schleswig-Holstein	PDA	-	[32]
**98**	*T. rubrum* Sin146 and *B. ochroleuca* Sin80	*T. rubrum* Sin146 was isolated from onychomycosis collected at the Centre Hospitalier Cantonal Vaudois.<br>*B. ochroleuca* Sin80 was collected as a soil isolate	PDA	-	[33]
**99**<br>**100**	*Cosmospora* sp. and *M. oryzae*	*Cosmospora* sp. was isolated from the Baltic Sea environment.<br>*M. oryzae* was obtained from Deutsche Sammlung für Mikroorganismen und Zellkulturen	PDA	-	[34]
**101**	*A. fischeri* NRRL 181 and *T. labelliformis* G536	*A. fischeri* NRRL 181 was acquired from the ARS Culture Collection. *T. labelliformis* G536 was isolated from surface sterilized twigs of paw paw (*A. triloba* (L.) Dunal, Annonaceae) collected from Pfafftown, NC, USA	Quaker Breakfast Oatmeal (10 g of oatmeal with 17 mL of deionized water)	-	[35]
**102**<br>**103**<br>**104**<br>**105**	*P. crustosum* PRB-2 and *Xylaria* sp. HDN13-249	*P. crustosum* PRB-2 was isolated from deep-sea sludge collected at a depth of 526 m in Prydz Bay<br>*Xylaria* sp. HDN13-249 was isolated from the root of Sonneratia caseolaris collected from the mangrove conservation area of Hainan, China	Solid medium (solublestarch 40 g/L, yeastextract 1 g/L, $MgSO_4$ 0.3 g/L, monosodium glutamate 2 g/L, sucrose 40 g/L, KH_2PO_4 0.5 g/L, maltose 30 g/L, bean flour 0.5g/L, peptone 2 g/L, agar power 25 g/L, 1 L seawater)	Compound **103** possessed potential antituberculosis effects.	[36]
**106**<br>**107**	*P. citrinum* MA-197 and *B. feline* EN-135.	*P. citrinum* MA-197 was isolated from the mangrove plant *Lumnitzera racemose*<br>*B. felina* EN-135 was isolated from an unidentified marine bryozoan	Wheat bran broth medium (100 mL of naturally sourced and fifiltered seawater from the Huiquan gulf of the Yellow Sea near the campus of IOCAS, 100 g of wheat bran, and 0.6 g of dried potato powder)	Inhibitory activities against several human and aquatic pathogens	[37]
**108**	*T. pinophilus* 17F4103 and *Paraphaeosphaeria* sp. 17F4110	Soil collected in Miyazaki Prefecture, Japan	Malt agar medium (malt extract 20 g/L, peptone 5 g/L, and agar 15 g/L 1 L deionized water)	**-**	[38]
**109**	*I. lacteus* 13S011 and *P. oryzae* 13S070.	Kumamoto Prefecture, Japan	Malt agar medium (malt extract 20 g/L, peptone 5 g/L, and agar 15 g/L 1 L deionized water)	**-**	[39]

Table S2 Fungal–bacterial co-culture derived new SMs (**110**–**151**).

Number	Co-culture Strains	Source	Co-culture Medium	Biological Activity	Reference
**110**	*S. terrestris* DBE-9 and *B. amyloliquifaciens* BBS4	*S. terrestris* DBE-9 was isolated from the leaf sample of *D. binectariferum* collected from the region of Bangalore, India *B. amyloliquifaciens* BBS4 was isolated from the soil sample	PDB	Cytotoxic effect on PC-3 and MCF-7 cell lines	[40]
**111** **112** **113** **114**	*Trichoderma* sp. (Strain 307) and *A. johnsonii* (Strain B2)	*Trichoderma* sp. (Strain 307) was Isolated from the stem bark of Clerodendrum inerme, collected in Zhanjiang Mangrove National Nature Reserve in Guangdong Province, China *A. johnsonii* (Strain B2) was isolated from an aquaculture pond at the Maoming Experimental Station in Guangdong, China	ISP2 broth	Compounds **113** and **114** exhibited potent α-glucosidase inhibitory activity.	[41]
**115** **116** **117** **118**	*Libertella* sp. CNL-523 and *α-proteobacterium* CNJ-328	*Libertella* sp. CNL-523 was isolated from an ascidian collected in the Bahamas α-proteobacterium CNJ-328 was isolated from a fungal culture	YPM medium (mannitol 4 g/L, yeast extract 2 g/L, peptone 2 g/L, 1 L seawater)	Cytotoxicity against the HCT-116 human adenocarcinoma cell line	[42]
**119** **120**	*A. fumigatus* and *S. peucetius*	*A. fumigatus* was acquired from the Michigan Department of Health. *S. peucetius* was purchased from the American Type Culture Collection.	ISP2 broth	-	[43]
**121**	*S. bullii* and *A. fumigatus* MBC-F1-10	*S. bullii* was isolated fromthe soil taken from the Laguna de Chaxa of the Salar de Atacama *A. fumigatus* MBC-F1-10 was isolated from the *S. bullii* culture	ISP2 broth	-	[44]
**122** **123**	*A. fumigatus* KMC-901 and *Sphingomonas sp.* KMK-001	An extremely contaminated acid mine drainage collected from a horizontal pit, situated at an elevation of 750 m at the Young-dong abandoned coal mine located at Gangneung, South Korea	Czapek-Dox broth (saccharose 30 g/L, sodium nitrate 3 g/L, dipotassium phosphate 1 g/L, magnesium sulfate 0.5 g/L, potassium chloride 0.5 g/L, ferrous sulfate 0.01 g/L, 1 L distilled H_2O)	-	[45,46]
**124** **125**	*P. aggolomerans* and *P. citrinum*	Laboratory of Soil Biology, University of Neuchatel, Switzerland	ISP2 broth	-	[47]
**126** **127** **128**	*F. pallidoroseum* ATCC 74289 and *S. erythraea* ATCC 31772	American Type Culture Collection (ATCC)	ISP2 broth	-	[48]

**129**<br>**130**	*Emericella sp.* CNL-878 and *S. Arenicola* CNH-665	*Emericella sp.* CNL-878 was isolated from the surface of a green alga of the genus *Halimeda*, collected at Madang Bay in Papua New Guinea. *S. Arenicola* CNH-665 was isolated from a sediment sample collected from the Bahamas	YPM medium (mannitol 4 g/L, yeast extract 2g/L, peptone 2 g/L, 1 L seawater)	Antibacterial activities	[49]
**131**	*Cladosporium* sp. WUH1 and *B. subtilis* CMCC (B) 63501	*B. subtilis* CMCC(B) 63501 was purchased from National Center for Medical Culture Collections. *Cladosporium* sp. WUH1 was isolated from hydrothermal vent sediment, collected from Kueishantao, Taiwan, China	PDA-LB liquid culture medium (potato lixivium 100 g/L, dextrose 10 g/L, yeast extract 5 g/L, peptone 10 g/L, NaCl 10 g/L)	Antibacterial activity	[50]
**132**<br>**133**<br>**134**<br>**135**<br>**136**	*F. tricinctum* and *S. lividans* TK24	*F. tricinctum* was obtained from healthyfresh rhizomes of *A. paucinervis* (Aristolochiaceae)	Rice medium	-	[51]
**137**<br>**138**	*A. versicolor* KU258497 and *B. subtilis* 168 trpC2	*A. versicolor* KU258497 was isolated from fresh healthy leaves of *Eichhornia crassipes*, family Pontederiaceae, obtained from the shores of the River Nile in Mansoura, Egypt	Rice medium	Compound 138 showed moderate cytotoxic activity against the mouse lymphoma cell line L5178Y	[52]
**139**<br>**140**<br>**141**<br>**142**	*A. sydowii* and *B. subtilis*	*A. sydowii* was isolated from a piece of deep-sea mud from Dalian, China	PDA	-	[53]
**143**	*Pestalotia* sp. CNL-365, and an unidentified, antibiotic-resistant marine bacterium	*Pestalotia* sp.CNL-365 from a sample of the brown alga *Rosenvingea* sp. collected in the Bahamas Islands	YPG Medium(glucose 10 g/L, peptone 5 g/L, yeast extract 5 g/L, agar 15 g/L, penicillinG 0.1g/L streptomycin sulfate 0.1 g/L, 1 L seawater)	Antibiotic activity against methicillin-resistant *S. aureus* and vancomycin-resistant *E. faecium*	[54]
**144**<br>**145**<br>**146**	*F. tricinctum* and *B. subtilis*168 trpC2.	*F. tricinctum* was isolated from fresh, healthy rhizomes of *Aristolochia paucinervis* collected from the mountains of Beni-Mellal Morocco	Rice medium	-	[55]
**147**	W. *actinomycete* WAC 2288 and *C. neoformans*	A Nigerian soil sample	Bennett's agar	Antimicrobial activity	[56]
**148**<br>**149**<br>**150**<br>**151**	*A. sversicolor* and *B. subtilis* 168 trpC2	*A. versicolor* was isolated from the sponge *Agelas oroides*, collected at a depth of 10 m in Aliağa-İzmir, Turkey	Rice medium	-	[57]

Table S3 Fungal–host co-culture derived new SMs (**152**–**158**).

Number	Co-culture Strain and Host	Source	Co-culture Medium	Biological Activity	Reference
**152**	*N. oryzae*, *I. lacteus*, and host plant *D. officinale*	*D.officinale* and *D. devoninum* seeds from Wenshan in Yunnan Province, China	PDB	Antifungal activity	[58]
**153**<br>**154**<br>**155**<br>**156**<br>**157**<br>**158**	*P. verruculosa* and the host plant *D. officinale*	*P. verruculosa* from the rhizosphere of *D. officinale* in Wenshan of Yunnan Province, China	PDB	-	[59]

References

1. Zhang, Z.X.; Yang, X.Q.; Zhou, Q.Y.; Wang, B.Y.; Hu, M.; Yang, Y.B.; Zhou, H.; Ding, Z.T. New azaphilones from *Nigrospora oryzae* co-cultured with *Beauveria bassiana*. *Molecules* **2018**, *23*, doi:10.3390/molecules23071816.
2. Li, H.-T.; Tang, L.-H.; Liu, T.; Yang, R.-N.; Yang, Y.-B.; Zhou, H.; Ding, Z.-T. Protoilludane-type sesquiterpenoids from *Armillaria* sp. by co-culture with the endophytic fungus *Epicoccum* sp. associated with *Gastrodia elata*. *Bioorg. Chem.* **2020**, *95*, doi:10.1016/j.bioorg.2019.103503.
3. Zhou, Q.-Y.; Yang, X.-Q.; Zhang, Z.-X.; Wang, B.-Y.; Hu, M.; Yang, Y.-B.; Zhou, H.; Ding, Z.-T. New azaphilones and tremulane sesquiterpene from endophytic *Nigrospora oryzae* cocultured with *Irpex lacteus*. *Fitoterapia* **2018**, *130*, 26-30, doi:10.1016/j.fitote.2018.07.018.
4. Wang, Z.-R.; Yang, H.-X.; Peng, X.-P.; Li, G.; Lou, H.-X. Induced production of zinniol analogues by co-cultivation of two endophytic fungi in the same ecological niche. *Phytochem. Lett.* **2020**, *35*, 206-210, doi:10.1016/j.phytol.2019.12.007.
5. Li, H.T.; Zhou, H.; Duan, R.T.; Li, H.Y.; Tang, L.H.; Yang, X.Q.; Yang, Y.B.; Ding, Z.T. Inducing secondary metabolite production by co-culture of the endophytic fungus *Phoma* sp. and the symbiotic fungus *Armillaria* sp. *J. Nat. Prod.* **2019**, *82*, 1009-1013, doi:10.1021/acs.jnatprod.8b00685.
6. Li, H.-T.; Liu, T.; Yang, R.; Xie, F.; Yang, Z.; Yang, Y.; Zhou, H.; Ding, Z.-T. Phomretones A-F, C-12 polyketides from the co-cultivation of *Phoma* sp. YUD17001 and *Armillaria* sp. *RSC. Adv.* **2020**, *10*, 18384-18389, doi:10.1039/d0ra02524k.
7. Stierle, A.A.; Stierle, D.B.; Decato, D.; Priestley, N.D.; Alverson, J.B.; Hoody, J.; McGrath, K.; Klepacki, D. The berkeleylactones, antibiotic macrolides from fungal coculture. *J. Nat. Prod.* **2017**, *80*, 1150-1160, doi:10.1021/acs.jnatprod.7b00133.
8. Vinale, F.; Nicoletti, R.; Borrelli, F.; Mangoni, A.; Parisi, O.A.; Marra, R.; Lombardi, N.; Lacatena, F.; Grauso, L.; Finizio, S.; et al. Co-culture of plant beneficial microbes as source of bioactive metabolites. *Sci. Rep.* **2017**, *7*, 14330, doi:10.1038/s41598-017-14569-5.
9. Gang Wang, H.R., Jie Fan, Nancy P. Keller, Zhiguo Liu, Fan Wu, Wen-Bing Yin. Fungal-fungal cocultivation leads to widespread secondary metabolite alteration requiring the partial loss-of-function VeA1 protein. *Sci. adv.* **2022**, *8*.
10. Liu, R.; Khan, R.A.A.; Yue, Q.; Jiao, Y.; Yang, Y.; Li, Y.; Xie, B. Discovery of a new antifungal lipopeptaibol from *Purpureocillium lilacinum* using MALDI-TOF-IMS. *Biochem. Bioph. Res. Co.* **2020**, *527*, 689-695, doi:10.1016/j.bbrc.2020.05.021.
11. Pham, H.T.; Doan, T.P.; Kim, H.W.; Kim, T.W.; Park, S.Y.; Kim, H.; Lee, M.; Kim, K.H.; Oh, W.K.; Lim, Y.W.; et al. Cyclohumulanoid sesquiterpenes induced by the noncompetitive coculture of *Phellinus orientoasiaticus* and *Xylodon flaviporus*. *J. Nat. Prod.* **2022**, *85*, 511-518, doi:10.1021/acs.jnatprod.1c01022.
12. Chen, J.X.; Xia, D.D.; Yang, X.Q.; Yang, Y.B.; Ding, Z.T. The antifeedant and antifungal cryptic metabolites isolated from tobacco endophytes induced by host medium and coculture. *Fitoterapia* **2022**, *163*, 105335, doi:10.1016/j.fitote.2022.105335.
13. Ge, X.; Wang, Y.; Sun, C.; Zhang, Z.; Song, L.; Tan, L.; Li, D.; Yang, S.; Yu, G. Secondary metabolites produced by coculture of *Pleurotus ostreatus* SY10 and *Pleurotus eryngii* SY302. *Chem. Biodivers.* **2022**, *19*, e202100832-n/a, doi:10.1002/cbdv.202100832.
14. Li, C.; Sarotti, A.M.; Yang, B.; Turkson, J.; Cao, S. A new N-methoxypyridone from the co-cultivation of hawaiian endophytic fungi *Camporesia sambuci* FT1061 and *Epicoccum sorghinum* FT1062. *Molecules* **2017**, *22*, doi:10.3390/molecules22071166.
15. Nonaka, K.; Abe, T.; Iwatsuki, M.; Mori, M.; Yamamoto, T.; Shiomi, K.; Omura, S.; Masuma, R. Enhancement of metabolites productivity of *Penicillium pinophilum* FKI-5653, by co-culture with *Trichoderma harzianum* FKI-5655. *J. Antibiot.* **2011**, *64*, 769-774, doi:10.1038/ja.2011.91.
16. Mandelare, P.E.; Adpressa, D.A.; Kaweesa, E.N.; Zakharov, L.N.; Loesgen, S. Coculture of two developmental stages of a marine-derived *Aspergillus alliaceus* results in the production of the cytotoxic bianthrone allianthrone A. *J. Nat. Prod.* **2018**, *81*, 1014-1022, doi:10.1021/acs.jnatprod.8b00024.
17. Shang, Z.; Salim, A.A.; Capon, R.J. Chaunopyran A: co-cultivation of marine mollusk-derived fungi activates a rare class of 2-alkenyl-tetrahydropyran. *J. Nat. Prod.* **2017**, *80*, 1167-1172, doi:10.1021/acs.jnatprod.7b00144.
18. Bao, J.; Wang, J.; Zhang, X.Y.; Nong, X.H.; Qi, S.H. New furanone derivatives and alkaloids from the co-culture of marine-derived fungi *Aspergillus sclerotiorum* and *Penicillium citrinum*. *Chem. Biodivers.* **2017**, *14*, doi:10.1002/cbdv.201600327.
19. Yao, L.; Zhu, L.P.; Xu, X.Y.; Tan, L.L.; Sadilek, M.; Fan, H.; Hu, B.; Shen, X.T.; Yang, J.; Qiao, B.; et al. Discovery of novel xylosides in co-culture of basidiomycetes *Trametes versicolor* and *Ganoderma applanatum* by integrated metabolomics and bioinformatics. *Sci. Rep.* **2016**, *6*, 33237, doi:10.1038/srep33237.
20. Zhu, F.; Li, J.-S.; Xie, W.-C.; Shi, J.-J.; Xu, F.; Song, Z.-F.; Liu, Y.-L. Structure revision of aspergicin by the crystal structure of aspergicine, a co-occurring isomer produced by co-culture of two mangrove epiphytic fungi. *Nat. Prod. Res.* **2017**, *31*, 2268-2272, doi:10.1080/14786419.2017.1300800.

21. Zhu, F.; Lin, Y.C. Marinamide, a novel alkaloid and its methyl ester produced by the application of mixed fermentation technique to two mangrove endophytic fungi from the South China Sea. *Chin. Sci. Bull.* **2006**, *51*, 1426-1430, doi:10.1007/s11434-006-1426-4.
22. Li, C.Y.; Ding, W.J.; Shao, C.L.; She, Z.G.; Lin, Y.C. A new diimide derivative from the co-culture broth of two mangrove fungi (strain no. E33 and K38). *J. Asian Nat. Prod. Res.* **2010**, *12*, 809-813, doi:10.1080/10286020.2010.497757.
23. Li, C.; Wang, J.; Luo, C.; Ding, W.; Cox, D.G. A new cyclopeptide with antifungal activity from the co-culture broth of two marine mangrove fungi. *Nat. Prod. Res.* **2014**, *28*, 616-621, doi:10.1080/14786419.2014.887074.
24. Huang, S.; Ding, W.; Li, C.; Cox, D.G. Two new cyclopeptides from the co-culture broth of two marine mangrove fungi and their antifungal activity. *Pharmacogn. Mag.* **2014**, *10*, 410-414, doi:10.4103/0973-1296.141781.
25. Zhang, Z.-X.; Yin, H.-Y.; Yang, Y.-B.; Wang, D.-L.; Zhao, T.-D.; Wang, C.-F.; Yang, X.-Q.; Ding, Z.-T. A new chlorinated tetralone from co-culture of insect-pathogenic *Beauveria bassiana* and phytopathogenic *Nigrospora oryzae*. *Chem. Nat. Compd+.* **2021**, *57*, 297-299, doi:10.1007/s10600-021-03343-9.
26. Li, F.; Yan, S.; Huang, Z.; Gao, W.; Zhang, S.; Mo, S.; Lin, S.; Wang, J.; Hu, Z.; Zhang, Y. Inducing new bioactive metabolites production from coculture of *Pestalotiopsis* sp. and *Penicillium bialowiezense*. *Bioorg. Chem.* **2021**, *110*, 104826, doi:10.1016/j.bioorg.2021.104826.
27. Sui-Qun Yanga, b., Xiao-Ming Lia, Xin Lia, Hong-Lei Lia, Ling-Hong Menga, Bin-Gui Wang. New citrinin analogues produced by coculture of the marine algal-derived endophytic fungal strains *Aspergillus sydowii* EN-534 and *Penicillium citrinum* EN-535. *Phytochem. Lett.* **2018**, *25*, 191-195, doi:10.1016/j.phytol.2018.04.023.
28. Meng, L.H.; Li, X.M.; Li, H.L.; Wang, B.G. Chermebilaenes A and B, new bioactive meroterpenoids from co-cultures of marine-derived isolates of *Penicillium bilaiae* MA-267 and *Penicillium chermesinum* EN-480. *Mar. Drugs* **2020**, *18*, doi:10.3390/md18070339.
29. Li, H.L.; Li, X.M.; Yang, S.Q.; Cao, J.; Li, Y.H.; Wang, B.G. Induced terreins production from marine red algal-derived endophytic fungus *Aspergillus terreus* EN-539 co-cultured with symbiotic fungus *Paecilomyces lilacinus* EN-531. *J. Antibiot.* **2020**, *73*, 108-111, doi:10.1038/s41429-019-0242-4.
30. Afiyatullov, S.S.; Zhuravleva, O.I.; Antonov, A.S.; Berdyshev, D.V.; Pivkin, M.V.; Denisenko, V.A.; Popov, R.S.; Gerasimenko, A.V.; von Amsberg, G.; Dyshlovoy, S.A.; et al. Prenylated indole alkaloids from co-culture of marine-derived fungi *Aspergillus sulphureus* and *Isaria felina*. *J. Antibiot.* **2018**, *71*, 846-853, doi:10.1038/s41429-018-0072-9.
31. Zhuravleva, O.I.; Belousova, E.B.; Oleinikova, G.K.; Antonov, A.S.; Khudyakova, Y.V.; Rasin, A.B.; Popov, R.S.; Menchinskaya, E.S.; Trinh, P.T.H.; Yurchenko, A.N.; et al. Cytotoxic drimane-type sesquiterpenes from co-culture of the marine-derived fungi *Aspergillus carneus* KMM 4638 and *Beauveria felina* (=*Isaria felina*) KMM 4639. *Mar. Drugs* **2022**, *20*, doi:10.3390/md20090584.
32. Oppong-Danquah, E.; Budnicka, P.; Blumel, M.; Tasdemir, D. Design of fungal co-cultivation based on comparative metabolomics and bioactivity for discovery of marine fungal agrochemicals. *Mar. Drugs* **2020**, *18*, doi:10.3390/md18020073.
33. Bertrand, S.; Schumpp, O.; Bohni, N.; Monod, M.; Gindro, K.; Wolfender, J.L. De novo production of metabolites by fungal co-culture of *Trichophyton rubrum* and *Bionectria ochroleuca*. *J. Nat. Prod.* **2013**, *76*, 1157-1165, doi:10.1021/np400258f.
34. Oppong-Danquah, E.; Blumel, M.; Scarpato, S.; Mangoni, A.; Tasdemir, D. Induction of Isochromanones by Co-Cultivation of the Marine Fungus Cosmospora sp. and the Phytopathogen Magnaporthe oryzae. *Int J Mol Sci* **2022**, *23*, doi:10.3390/ijms23020782.
35. Knowles, S.L.; Raja, H.A.; Isawi, I.H.; Flores-Bocanegra, L.; Reggio, P.H.; Pearce, C.J.; Burdette, J.E.; Rokas, A.; Oberlies, N.H. Wheldone: Characterization of a unique scaffold from the coculture of *Aspergillus fischeri* and *Xylaria flabelliformis*. *Org. Lett.* **2020**, *22*, 1878-1882, doi:10.1021/acs.orglett.0c00219.
36. Yu, G.; Sun, Z.; Peng, J.; Zhu, M.; Che, Q.; Zhang, G.; Zhu, T.; Gu, Q.; Li, D. Secondary metabolites produced by combined culture of *Penicillium crustosum* and a *Xylaria* sp. *J. Nat. Prod.* **2019**, *82*, 2013-2017, doi:10.1021/acs.jnatprod.9b00345.
37. Meng, L.H.; Liu, Y.; Li, X.M.; Xu, G.M.; Ji, N.Y.; Wang, B.G. Citrifelins A and B, citrinin adducts with a tetracyclic framework from cocultures of marine-derived isolates of *Penicillium citrinum* and *Beauveria felina*. *J. Nat. Prod.* **2015**, *78*, 2301-2305, doi:10.1021/acs.jnatprod.5b00450.
38. Murakami, S.; Hayashi, N.; Inomata, T.; Kato, H.; Hitora, Y.; Tsukamoto, S. Induction of secondary metabolite production by fungal co-culture of *Talaromyces pinophilus* and *Paraphaeosphaeria* sp. *J. Nat. Med.* **2020**, *74*, 545-549, doi:10.1007/s11418-020-01400-1.
39. Sadahiro, Y.; Kato, H.; Williams, R.M.; Tsukamoto, S. Irpexine, an isoindolinone alkaloid produced by coculture of endophytic fungi, *Irpex lacteus* and *Phaeosphaeria oryzae*. *J. Nat. Prod.* **2020**, *83*, 1368-1373, doi:10.1021/acs.jnatprod.0c00047.
40. Arora, D.; Chashoo, G.; Singamaneni, V.; Sharma, N.; Gupta, P.; Jaglan, S. *Bacillus amyloliquefaciens* induces production of a novel blennolide K in coculture of *Setophoma terrestris*. *J. Appl. Microbiol.* **2018**, *124*, 730-739, doi:10.1111/jam.13683.

41. Zhang, L.; Niaz, S.I.; Khan, D.; Wang, Z.; Zhu, Y.; Zhou, H.; Lin, Y.; Li, J.; Liu, L. Induction of diverse bioactive secondary metabolites from the mangrove endophytic fungus *Trichoderma* sp. (Strain 307) by co-cultivation with *Acinetobacter johnsonii* (Strain B2). *Mar. Drugs* **2017**, *15*, doi:10.3390/md15020035.
42. Oh, D.C.; Jensen, P.R.; Kauffman, C.A.; Fenical, W. Libertellenones A-D: induction of cytotoxic diterpenoid biosynthesis by marine microbial competition. *Bioorg. Med. Chem.* **2005**, *13*, 5267-5273, doi:10.1016/j.bmc.2005.05.068.
43. Zuck, K.M.; Shipley, S.; Newman, D.J. Induced production of N-formyl alkaloids from *Aspergillus fumigatus* by co-culture with *Streptomyces peucetius*. *J. Nat. Prod.* **2011**, *74*, 1653-1657, doi:10.1021/np200255f.
44. Rateb, M.E.; Hallyburton, I.; Houssen, W.E.; Bull, A.T.; Goodfellow, M.; Santhanam, R.; Jaspars, M.; Ebel, R. Induction of diverse secondary metabolites in *Aspergillus fumigatus* by microbial co-culture. *RSC Adv.* **2013**, *3*, doi:10.1039/c3ra42378f.
45. Hyun Bong Park, H.C.K., Chang-Ho Lee, and Hyun Ok Yang. Glionitrin A, an antibioic-antitumor metabolite derived from competitive interaction between abandoned mine microbes. *J. Nat. Prod* **2009**, *72*, 248–252.
46. Park, H.B.; Kim, Y.J.; Park, J.S.; Yang, H.O.; Lee, K.R.; Kwon, H.C. Glionitrin B, a cancer invasion inhibitory diketopiperazine produced by microbial coculture. *J. Nat. Prod.* **2011**, *74*, 2309-2312, doi:10.1021/np200563x.
47. Thissera, B.; Alhadrami, H.A.; Hassan, M.H.A.; Hassan, H.M.; Bawazeer, M.; Yaseen, M.; Belbahri, L.; Rateb, M.E.; Behery, F.A. Induction of cryptic antifungal pulicatin derivatives from pantoea agglomerans by microbial co-culture. *Biomolecules* **2020**, *10*, doi:10.3390/biom10020268.
48. Whitt, J.; Shipley, S.M.; Newman, D.J.; Zuck, K.M. Tetramic acid analogues produced by coculture of *Saccharopolyspora erythraea* with *Fusarium pallidoroseum*. *J. Nat. Prod.* **2014**, *77*, 173-177, doi:10.1021/np400761g.
49. Dong-Chan Oh, C.A.K., Paul R. Jensen, and William Fenical. Induced production of emericellamides A and B from the marine derived fungus *Emericella* sp. in competing coculture. *J. Nat. Prod.* **2007**, *70*, 515-520.
50. Shi, Y.; Pan, C.; Wang, K.; Chen, X.; Wu, X.; Chen, C.A.; Wu, B. Synthetic multispecies microbial communities reveals shifts in secondary metabolism and facilitates cryptic natural product discovery. *Environ. Microbiol.* **2017**, *19*, 3606-3618, doi:10.1111/1462-2920.13858.
51. Moussa, M.; Ebrahim, W.; Bonus, M.; Gohlke, H.; Mandi, A.; Kurtan, T.; Hartmann, R.; Kalscheuer, R.; Lin, W.; Liu, Z.; et al. Co-culture of the fungus *Fusarium tricinctum* with *Streptomyces lividans* induces production of cryptic naphthoquinone dimers. *RSC. Adv.* **2019**, *9*, 1491-1500, doi:10.1039/c8ra09067j.
52. Abdelwahab, M.F.; Kurtán, T.; Mándi, A.; Müller, W.E.G.; Fouad, M.A.; Kamel, M.S.; Liu, Z.; Ebrahim, W.; Daletos, G.; Proksch, P. Induced secondary metabolites from the endophytic fungus *Aspergillus versicolor* through bacterial co-culture and OSMAC approaches. *Tetrahedron Lett* **2018**, *59*, 2647-2652, doi:10.1016/j.tetlet.2018.05.067.
53. Sun, Y.; Liu, W.C.; Shi, X.; Zheng, H.Z.; Zheng, Z.H.; Lu, X.H.; Xing, Y.; Ji, K.; Liu, M.; Dong, Y.S. Inducing secondary metabolite production of *Aspergillus sydowii* through microbial co-culture with *Bacillus subtilis*. *Microb. Cell. Fact.* **2021**, *20*, 42, doi:10.1186/s12934-021-01527-0.
54. Mercedes Cueto, P.R.J.e., Chris Kauffman, William Fenical,Emil Lobkovsky, Jon Clardy. Pestalone , a new antibiotic produced by a marine fungus in response to bacterial challenge. *J. Nat. Prod.* **2001**, 64, 1444-1446.
55. Ola, A.R.B.; Thomy, D.; Lai, D.; Brötz-Oesterhelt, H.; Proksch, P. Inducing secondary metabolite production by the endophytic fungus *Fusarium tricinctum* through coculture with *Bacillus subtilis*. *J. Nat. Prod.* **2013**, *76*, 2094-2099, doi:10.1021/np400589h.
56. Robbins, N.; Spitzer, M.; Wang, W.; Waglechner, N.; Patel, D.J.; O'Brien, J.S.; Ejim, L.; Ejim, O.; Tyers, M.; Wright, G.D. Discovery of Ibomycin, a complex macrolactone that exerts antifungal activity by impeding endocytic trafficking and membrane function. *Cell Chem. Biol.* **2016**, *23*, 1383-1394, doi:10.1016/j.chembiol.2016.08.015.
57. Abdel-Wahab, N.M.; Scharf, S.; Ozkaya, F.C.; Kurtan, T.; Mandi, A.; Fouad, M.A.; Kamel, M.S.; Muller, W.E.G.; Kalscheuer, R.; Lin, W.; et al. Induction of secondary metabolites from the marine-derived fungus *Aspergillus versicolor* through co-cultivation with *Bacillus subtilis*. *Planta Med.* **2019**, *85*, 503-512, doi:10.1055/a-0835-2332.
58. Shi, L.-J.; Wu, Y.-M.; Yang, X.-Q.; Xu, T.-T.; Yang, S.; Wang, X.-Y.; Yang, Y.-B.; Ding, Z.-T. The cocultured *Nigrospora oryzae* and *Collectotrichum gloeosporioides*, *Irpex lacteus*, and the plant host *dendrobium officinale* bidirectionally regulate the production of phytotoxins by anti-phytopathogenic metabolites. *J.Nat. Prod.* **2020**, *83*, 1374-1382, doi:10.1021/acs.jnatprod.0c00036.
59. Hu, M.; Yang, X.Q.; Wang, C.F.; Zhao, T.D.; Wang, D.L.; Yang, Y.B.; Ding, Z.T. Paraverrucsins A-F, antifeedant, and antiphytopathogenic polyketides from rhizospheric *paraphaeosphaeria verruculosa* and induced bioactivity enhancement by coculturing with host plant *Dendrobium officinale*. *ACS Omega.* **2020**, *5*, 30596-30602, doi:10.1021/acsomega.0c04548.
